# Posttraumatic stress in German volunteer lifeguards: evidence for the building block effect

**DOI:** 10.1186/s12889-026-28126-1

**Published:** 2026-07-02

**Authors:** Kim Madeleine Stucke, Kai Jannik Nehler, Martin Schultze

**Affiliations:** 1 Department of Child and Adolescent Psychiatry, Psychosomatics and Psychotherapy, Ulm University, Ulm, Germany; 2 German Center for Mental Health (DZGP), Partner Site Mannheim-Heidelberg-Ulm, Ulm, Germany; 3 Department of Psychology, Goethe University Frankfurt, Frankfurt am Main, Germany; 4 Department of Psychology, HMU Health and Medical University Erfurt, Erfurt, Germany

**Keywords:** Emergency responders, Posttraumatic stress symptoms, Lifeguards, Volunteers

## Abstract

**Background:**

Emergency responders are regularly exposed to potentially traumatic events, placing them at risk of developing posttraumatic stress symptoms (PTSS). Within this group, lifeguards remain an understudied population. The present study examined the building block effect in volunteer emergency responders of the German Lifesaving Association (DLRG), hypothesizing that a higher number of stressful events would be associated with greater PTSS. In addition, potential influencing factors on symptom severity and on the relationship between event exposure and PTSS, including gender, age, perceived social support, and peer support measures, were investigated.

**Method:**

Data were collected via a web-based self-report survey, resulting in a final analysis sample of 365 lifeguards from the DLRG. Participants were nested within 198 local subgroups. Generalized linear mixed models were constructed, with predictors entered in a stepwise manner.

**Results:**

The findings support the building block effect of stressful experiences, indicating that greater exposure is associated with higher PTSS among lifeguards in Germany. Hypothesis testing further revealed protective effects of higher age, male gender, greater perceived social support, and stronger peer support measures in reducing symptom severity. Interaction effects of these protective factors on the relationship between the number of traumatic events and PTSS were non-significant. Exploratory analyses also suggested that higher rank within the organization may be associated with lower symptom severity.

**Conclusion:**

The study highlights the importance of addressing mental health challenges among volunteer lifeguards, acknowledging that PTSS do not necessarily stem from a single worst traumatic event but can develop cumulatively. The findings also indicate target groups for additional training efforts, particularly younger women, and underscore the value of fostering support networks.

**Supplementary Information:**

The online version contains supplementary material available at 10.1186/s12889-026-28126-1.

## Introduction

Emergency responders are known to be at high risk for mental disorders, particularly posttraumatic stress disorder (PTSD) [1], a mental health condition that can develop after exposure to traumatic events and is characterized by persistent distress and functional impairment [2]. Studies from various countries have shown elevated rates of PTSD [3] and subclinical levels of posttraumatic stress symptoms (PTSS) among emergency responders [4, 5]. Research also suggests that prevalence rates and underlying mechanisms cannot be generalized across occupational groups [1, 6, 7]. While firefighters have been studied extensively [8–10], lifeguards remain significantly understudied, with only two published studies to date [11, 12]. Even within the group of lifeguards, there are organizational differences, such as employment status and tasks, that may influence posttraumatic stress outcomes [13]. The present study focuses on posttraumatic stress in lifeguards in Germany, all of whom are engaged on a voluntary basis.

### Lifeguards in Germany

As lifeguards provide services during various critical events, they can be classified as emergency responders [14]. In Germany, lifeguards^1^ are primarily affiliated with the German Life Saving Association (“Deutsche Lebens-Rettungs-Gesellschaft, DLRG”). The DLRG is the largest voluntary water rescue organization in the world and its overarching mission is to prevent fatalities from drowning [15]. The approximately 70,000 actively involved emergency responders, with a minimum age of 16 years, are organized into over 2,000 local groups distributed across Germany [16]. As stated earlier, all emergency responders in the DLRG serve as volunteers. While the organization comprises multiple departments, emergency responses are primarily handled by two: the water rescue service and the Civil Protection Department. The latter includes emergency responders deployed in disaster control (e.g., during floods) as well as rapid response groups that can be activated to respond to emergencies or missing people on waterways. At the German coast, the water rescue service deploys around 4,000 lifeguards during the summer season to supervise bathing areas, prevent water-related hazards, provide first aid, and search for missing persons [17]. The significance of these responsibilities is underscored by the fact that in 2023, the DLRG was called upon to aid in approximately 60,000 instances, of which more than 1,000 involved life-saving interventions [18].

### PTSD and the building block effect

The Diagnostic and Statistical Manual of Mental Disorders (DSM-5) [2] defines PTSD as a condition characterized by intrusive memories of the traumatic event(s), dissociative reactions, persistent avoidance behaviors, and negative alterations in cognitions. Criterion A for PTSD diagnosis, one of several criteria that must be met according to the DSM-5, requires exposure to trauma, which may be direct or indirect. Direct exposure is defined as personally experiencing the traumatic event, whereas indirect exposure is defined as witnessing the event as it occurs to others or learning that it happened to a close family member or friend. The DSM-5 further specifies that work as an emergency responder can fulfill this criterion [19]. Trauma exposure in the context of emergency response is particularly relevant for lifeguards, who are regularly involved in emergency situations such as drowning incidents or medical emergencies, which constitute indirect exposure. In addition, their frequent work in hazardous water conditions can be considered a form of direct exposure.

The DSM-5 also specifies that individuals may be exposed to traumatic events through different forms of exposure and across repeated experiences [2]. Wilker et al. [19] refer to the resulting increase in PTSD risk and symptom severity due to repeated exposure as the *building block effect*. Conceptually, the building block effect reflects a dose-response relationship, describing a cumulative association between lifetime PTEs and adverse mental health outcomes [20]. This is supported by several studies demonstrating that higher levels of traumatic exposure are linked to increased PTSD prevalence [21–23] and more severe PTSS [24, 25]. Evidence from occupational settings further supports this notion: for example, in professional firefighters, repeated (including indirect) exposure to traumatic events in daily work has been associated with the occurrence of PTSS [10]. In the context of volunteer lifeguards who also engage in regular employment and everyday activities, PTEs may also arise outside of their voluntary work [26] and represent an additional source of cumulative risk for PTSS.

### Influencing factors

Beyond exposure to trauma, other factors are associated with PTSS. Sociodemographic variables such as gender and age have been widely studied. Research has established that PTSD has a two-to-one ratio in the general population, with women exhibiting a heightened risk of developing the condition, even though the basis of these differences is less well-established [27, 28]. Proposed explanations include biological factors such as genetic predispositions and hormonal influences [27]. In the context of emergency responders, Soravia et al. [29] reported that female firefighters were associated with an increase in PTSS and Carmassi et al. [30] found that female emergency healthcare workers were more prone to develop PTSS. Age has also been investigated in this population, with younger firefighters found to exhibit higher levels of PTSS than older ones [6], a pattern that is supported by reviews of several studies on different types of emergency responders [3, 31] and aligns with findings from general population samples [28]. The reasons underlying this association remain unclear, although young adulthood and adolescence are generally considered to be periods of heightened risk for mental health problems [31], whereas older individuals have been reported to show greater resilience and stronger cognitive reappraisal capacities [28, 32, 33].

Regarding the occupational context, findings are mixed. Some studies have reported lower PTSS scores among volunteer emergency responders compared to full-time personnel [6, 34]. In contrast, other studies have found higher PTSS levels in volunteers than in professionals [7, 35], leaving the impact of volunteering uncertain. Potential explanations may include differences in recruitment and screening procedures [36], increased work-family conflicts in volunteers [37], or access to organizational support [38]. Research on the influence of rank, understood as an individual’s hierarchical position or role within the operational chain of command, has also produced inconclusive results. It could be assumed that rank may influence PTSS through differences in leadership responsibility, decision-making pressure, exposure to critical incidents, and workload during emergency operations. While one study found that individuals in lower ranks (e.g., non-commanding roles) were more likely to experience PTSS [39], another reported higher PTSS levels among those in commanding roles, such as firefighter commanders [6].

Perceived social support, referring to an individual’s perception of available informal support from one’s social environment, has also been widely studied in the context of trauma exposure. Low levels of social support have been associated with an increased risk of developing PTSS [39, 40], while a high level of social support appears to act as a protective factor [3, 41, 42]. Peer support measures differ from social support in that they are structured interventions typically led by trained individuals. Peer support in the DLRG refers to psychological emergency care provided by qualified peers, which may include structured debriefings and individual supportive conversations [43]. Peer support interventions have been described as helpful in qualitative studies [41, 44].

Theoretical knowledge in the form of psychoeducation on self-care, including information about risk factors and available support, may play a protective role in the development of PTSS among emergency responders. Lanza et al. [45] demonstrated empirically that a comprehensive prevention program containing such content had positive effects on mental health. In addition, they highlighted through theoretical vignettes that psychoeducation may also help emergency responders cope with potentially traumatic events and support later treatment processes.

To our knowledge, only two studies have investigated the effects of lifeguarding on the development of PTSS. The first study, conducted in New Zealand, examined personal and surf lifesaving trauma exposure, PTSS, posttraumatic growth, perceived social support, and self-efficacy [12]. Key findings indicated that young lifeguards, some starting as early as age 14, may be more vulnerable to PTSS. Female lifeguards also showed higher PTSS scores, and a significant relationship between trauma exposure and PTSS in lifeguards was found. The second study, conducted in Australia, involved a small sample of volunteer surf lifesavers and paid lifeguards [11]. It explored differences between adolescents and adults, as well as gender and social support. A significant positive relationship between direct trauma exposure and PTSS was found among adolescents, aligning with the findings of Rooke and de Terte [12], who reported higher PTSS levels in younger participants.

### Goal of study and hypotheses

Lifeguards are frequently exposed to potentially traumatic events, creating a context that allows for the investigation of the building block effect. While the relationship between stressful events and various influencing factors has been examined in different contexts [7, 9, 42], research specifically focusing on lifeguards remains scarce. Existing studies on PTSS in lifeguards have primarily focused on bivariate associations [11, 12]. In the present study, we aim to investigate the effects of different theoretically derived covariates within a joint model in the context of the building block effect. Furthermore, in contrast to the populations investigated in previous studies [11, 12], all lifeguards in the DLRG in Germany are volunteers. This may lead to differing results [34], although it also means that this factor cannot be varied within the current sample. Combined with the unique responsibilities of German lifeguards — including rapid response, disaster control operations, and water rescue services — this constitutes a previously unexplored population for research.

The main hypothesis is that the building block effect [19] is also present in DLRG lifeguards. This would mean that lifeguards who have encountered a larger number of stressful events will exhibit stronger PTSS (H1). However, since the sample will only include volunteer lifeguards, participants will also have regular employment. Stressful events may therefore also occur in their professional or personal lives. We aim to investigate this separately and propose a second hypothesis for the building block effect — that more stressful events will lead to higher PTSS — in the context of events outside the DLRG (H2). This variable will thereby also serve as a control for potential confounding, given that the volunteer work typically occupies only a limited proportion of an individual’s overall life context.

Given the established elevated risk for PTSD among females in the general population [28], it was hypothesized that female lifeguards would exhibit higher levels of PTSS compared to male lifeguards (H3a). Based on studies of emergency responders, it was further postulated that female lifeguards would also exhibit a more pronounced association between stressful events and PTSS compared to male lifeguards (H3b) [29, 30].

Age also warrants consideration, as younger emergency responders have been shown to be more vulnerable to stress-related disorders than older ones (H4a) [3, 6, 31]. Moreover, the relationship between exposure to stressful events and PTSS should be stronger among younger lifeguards (H4b) [12].

Previous research suggests that strong perceived social support mitigates the relationship between stressful events and PTSS, thereby exerting a buffering effect (H5) [3, 11, 41, 42]. Given the clear objectives, it is also hypothesized that emergency responders who participate in peer support measures will exhibit lower PTSS in response to stressful events than those who do not (H6) [41, 44]. Notably, in contrast to social support, which is conceptualized in terms of perceived availability, our focus regarding peer support lies on the frequency of participation in such measures.

Exploratory testing is conducted to examine potential influencing factors in areas where prior evidence is inconclusive or not readily transferable to the specific context of the lifeguards examined in this study. As previously noted, findings on the role of rank among emergency responders have been mixed [6, 39]. Additionally, rank structures and their psychological relevance may vary considerably between lifeguards and, for example, military personnel. Furthermore, theoretical knowledge is also examined as a potential influencing factor in the exploratory analyses. A positive effect might be expected based on the descriptions provided by Lanza et al. [45]. However, the measure of theoretical knowledge in the present study does not reflect participation in a structured psychoeducational course but is based on a cross-sectional self-report. As such, it remains unclear when the reported knowledge was acquired — that is, whether it preceded, followed shortly after, or occurred long after exposure to potentially traumatic events. In the latter case, a preventive effect would be less likely. Moreover, since psychoeducational content has only recently been integrated into DLRG training programs, the reported knowledge may not necessarily reflect participation in such standardized formats, especially among older lifeguards with longer service histories.

## Methods

The study’s a priori hypotheses and planned methods were preregistered on the *Open Science Framework* (OSF)^2^. Data were collected via an online self-report survey distributed to DLRG emergency responders through social media and mailing lists. As outlined in the preregistration, no power analysis was conducted due to the lack of prior research using comparable analytical models. The objective was to maximize the number of observations within the available timeframe. Overall, 439 participants initiated the survey, which was accessible for two months. As preregistered, individuals without sufficient proficiency in German or without current active engagement as emergency responders were removed from the dataset. Moreover, participants who selected “diverse” as their gender were not included, as their number did not reach the predefined threshold of 50 cases required for reliable estimation of the group-specific parameters, leaving 425 participants. Contrary to the preregistration, participants with missing values on the dependent variable were excluded due to the absence of auxiliary variables for imputing missing data. The final dataset therefore consisted of *N* = 365 participants. An attrition analysis comparing excluded and retained participants showed no significant differences in the means or variances of reported potentially traumatic events. Participation was voluntary, and informed consent was obtained at the start of the survey. No financial compensation or other incentives were offered. The study was approved by the local ethics committee of the Institute of Psychology and Sports Sciences at Goethe University Frankfurt, Germany, in July 2024 (File 2024-32).

### Participant characteristics

Participants in the final analysis sample had a mean age of *M* = 29.85 years (*SD* = 12.28) and had been actively involved in the DLRG for an average of *M* = 11.53 years (*SD* = 10.91), with the two variables showing a strong positive correlation (*r* =.80). Participants ranged in age from 16 to 75 years, with active involvement spanning 1 to 60 years. Twice as many male emergency responders (*n* = 244) as female emergency responders participated. The participants originated from all German federal state associations, although the distribution was not uniform. Representativeness is difficult to assess, as the state associations vary in size and no centralized access to this information exists. Most participants indicated that they were active in both areas surveyed (55.34%). A smaller proportion were solely involved in the water rescue service (30.13%), while fewer were exclusively engaged in the Civil Protection Department (14.52%).

### Measures

#### Sociodemographic measures

Participants reported their age, gender, the federal state in which they were active, and their local group affiliation. Additionally, the number of active years as an emergency responder was recorded. Participants indicated the rank they held for most of their time in service by selecting one of the following: on-site emergency responder, team leader (“Truppführer*in”), group leader (“Gruppenführer*in”), station leader (“Wachführer*in”), deputy operations leader (“Zugführer*in”), or operations leader. All questionnaire items and response options reported for illustrative purposes in the following sections are English translations of the German versions used in this study.

#### Stressful events

The number of stressful events experienced by the emergency responders in the past was measured using a modified version of the *Life Events Checklist for DSM-5* (LEC-5), German Version [46]. To better reflect the experiences of lifeguards, two items were added to the checklist and one was modified, following the adaptation approach of Fien et al. [11]. Specifically, the items “rescue attempt of people in need of help” and “recovery of bodies” were added, and “drowning” was included as an example under “sudden violent death”, resulting in a total of 19 items. Participants indicated the frequency with which they had experienced specific events separately for experiences within and outside the DLRG, using the following response options: never, 1–2 times, 3–5 times, 5–10 times, and more than 10 times. This response format, proposed by Wilker et al. [19], was successfully employed in previous research [47]. It assumes that exact frequencies cannot be accurately recalled, particularly for larger numbers, due to the trauma memory structure. In addition to the 19-item checklist, the LEC-5 includes a second part with two open-ended questions, allowing participants to report additional events they have experienced and to elaborate on the one they personally perceived as the most distressing, along with eight additional questions about that event.

#### Posttraumatic stress symptoms

The severity of PTSS was measured using the German version of the *PTSD Checklist for DSM-5* (PCL-5), which consists of 20 items rated on a five-point Likert scale ranging from 0 (“not at all”) to 4 (“extremely”) [48]. The PCL-5 was chosen for its alignment with DSM-5 criteria for PTSD, its widespread use in clinical and research settings [49], and its purpose of quantifying PTSS severity [50]. The original version of the instrument has demonstrated high internal consistency (α = 0.95), high test-retest reliability (*r* =.91), and good convergent validity [48]. In the present study, we modified the wording of all items in the PCL-5 so that they did not refer to a single most distressing event, but rather to all traumatic events reported previously in the LEC-5. The modification did not affect the internal consistency of the scale, which was also high (Cronbach’s α = 0.94).

#### Perceived social support, peer support and theoretical knowledge

Perceived social support was measured using the German Version of the *ENRICHD Social Support Inventory* (ESSI) [51]. It contains five items rated on a five-point Likert scale ranging from 0 (“never”) to 4 (“always”). The psychometric characteristics of the questionnaire are deemed to be satisfactory, with Cronbach’s α = 0.89 in the original validation [51], as well as in the present study, where Cronbach’s α was high at 0.92.

Peer support was measured using the item “After one or more stressful events within the DLRG, did peer support take place?”. Responses were rated on a five-point Likert scale ranging from 0 (“never”) to 4 (“always”), indicating frequency.

Theoretical knowledge was assessed using the item “Have you acquired theoretical knowledge about dealing with mental stress through your professional or internal DLRG training?”, with response options “yes” and “no”. This wording does not specifically require that this knowledge was gained through the DLRG, as this type of education has been integrated into the organization’s curriculum in recent years, but DLRG volunteers may also acquire psychological knowledge through their primary occupations.

### Statistical analyses and software

All analyses were conducted using R [52] (Version 4.5.2). Descriptive statistics were calculated using the packages *psych *[53] (Version 2.5.6) and *rococo *[54] (Version 1.1.9). DLRG ranks were grouped into three categories in consultation with an advisor familiar with the organizational structure. The *low* category comprises operational roles involving direct contact with patients and incident scenes (on-site emergency responder, team leader). The *middle* category includes mid-level leadership positions responsible for communication and transmission of orders (group leader, station leader). The *high* category covers top-level leadership roles with overall responsibility for operations (deputy operations leader, operations leader). In line with Wilker et al. [19], LEC responses for events experienced within and outside the DLRG were coded on a scale from 0 to 4: 0 = never, 1 = 1–2 times, 2 = 3–5 times, 3 = 5–10 times, and 4 = more than 10 times. Two frequency scores for traumatic events were computed by summing the responses to the LEC-5 items for events inside and outside the DLRG. A sum score for the ESSI was calculated to reflect perceived social support. The symptom severity score, calculated as the sum of all PCL-5 items, served as an indicator of PTSS severity.

The data were modeled using a two-level structure, assuming that lifeguards within the same local group would exhibit greater similarity than those in different local groups. This structure reflects potential variations in operational procedures and in the nature of the operations themselves. The 365 emergency responders were nested within 198 local groups. While group-mean centering is generally recommended when modeling interactions in hierarchical data [55, 56], all non-categorical predictors involved in interactions were centered on the grand mean (including perceived social support, contrary to the preregistration). This choice was made because nearly all local groups included fewer than four observations, so group-mean centering would assign a value of zero to many observations, potentially introducing substantial bias in the estimation of Level-1 effects, which are central to our analyses. In contrast, the bias introduced by grand-mean centering is considered to be smaller in this context.

The initial step involved checking the assumptions of a linear mixed model using the *lme4* package [57] (Version 1.1.37). While the intraclass correlation coefficient (ICC) calculation using the *ICC* package [58] (Version 2.4.0) indicated the need for a multilevel structure, the distribution of the dependent variable strongly violated the assumption of normally distributed residuals. An attempt to transform the dependent variable to approximate normality using a Box-Cox transformation as implemented in the *MASS* package [59] (Version 7.3.65), as specified in the preregistration, was unsuccessful. As an alternative, generalized linear mixed models (GLMMs) were used. A zero-inflated negative binomial model was selected to account for the right-skewed distribution of the data, which included a substantial number of responses indicating the absence of symptoms. This model is particularly suited for clinical data containing both structural zeros — that is, a true absence of symptoms — and sampling zeros, which occur due to sampling variability [60]. In our analysis, the zero-inflation component was specified without predictors, as no hypotheses were formulated in the preregistration of the study about the factors driving structural absence of symptoms. Instead, all predictors were tested in the count component, which estimates the severity of symptoms among individuals who show at least some symptom expression. The resulting regression coefficients reflect the association between the predictors and symptom severity in the subgroup of individuals who are not structurally symptom-free. Importantly, these associations are modeled on the log scale, meaning that the coefficients represent multiplicative, rather than additive, changes in expected symptom counts. The negative binomial two distribution was selected for the GLMMs in accordance with the Bayesian Information Criterion (BIC).

GLMMs were fitted using the *glmmTMB* package [61] (Version 1.1.12). ICC for GLMMs and pseudo-*R²* values were obtained via the *performance *[62] package (Version 0.15.0). Within this calculation, marginal and conditional pseudo-*R²* values are obtained [63]. Assumptions for each of the models described below were tested using the *performance* and the *DHARMa *[64] (Version 0.4.7) packages. All assumptions were met, except for the normality of random effects. This violation is considered acceptable, as it reduces the precision of the random effects estimates [65], which were not the focus of the present study. A commented documentation of the preliminary tests for the GLMM models, assumption tests and the full analytical procedure is available on the OSF^3^.

**Fig. 1:**
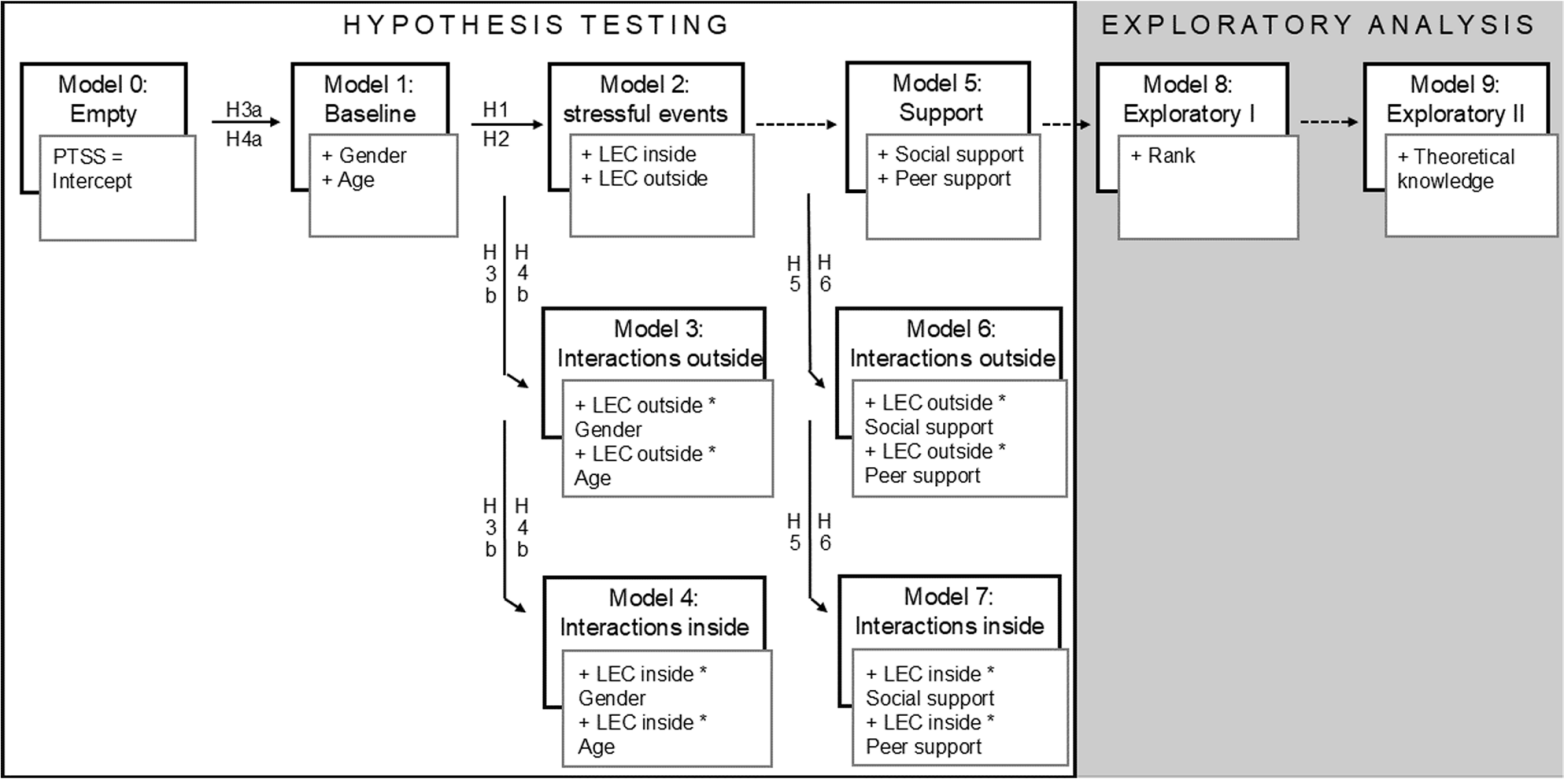
Flowchart stepwise model-building approach. *Note*: Models 0–7 represent hypothesis testing, with predictors added stepwise according to H1–H6. Models 8–9 are exploratory, adding variables without prior hypotheses. “LEC inside” and “LEC outside” denote stressful events inside or outside the DLRG context

To investigate the factors influencing PTSS, a stepwise model-building approach was used, incrementally adding predictors and interaction terms based on the study’s hypotheses (see Fig. 1). Model 0 served as the intercept-only baseline model. Random intercepts were included in all models. In Model 1, well-established sociodemographic predictors (age and gender) were included. Model 2 added stressful events occurring inside and outside the DLRG, followed by Models 3 and 4, which introduced their interactions with sociodemographic predictors. Model 5 included social support and peer support as predictors. As preregistered, random slopes were considered only for these two variables. Models 6 and 7 added interactions between the support variables and the stressful events inside and outside the DLRG. Non-significant terms were excluded in subsequent steps based on $\:{{\upchi\:}}^{2}$ model comparisons.

Exploratory variables were integrated into this framework in two additional steps. In Model 8, the variable rank (levels: low, middle, high) was added using dummy coding with middle rank as the reference category. This choice allowed for indirect comparisons between high and low rank. In Model 9, theoretical knowledge was added as a predictor. Model comparisons in the exploratory steps were also conducted using $\:{{\upchi\:}}^{2}$ model comparisons. However, these should not be interpreted as formal hypothesis tests; rather, they served to identify the best-fitting model within the current data, with the aim of enabling hypothesis generation for future research.

Since no power analysis was conducted, the *bayestestR* package [66] (Version 0.16.1) was used to compute an approximation of the Bayes factor (BF) [67] in addition to significance-based model comparisons. The interpretation of the BF follows the classification proposed by Raftery [68]. However, the decision to retain predictors and interactions in the model was solely based on the *p*-value.

## Results

### Descriptive analysis

Regarding PTSS (PCL-5), a large proportion of observations (24.38%) had a score of 0, as described earlier. Among the non-zero observations, the mean score was *M* = 9.95 (*SD* = 11.15). The full distribution is shown in the Electronic Supplementary Material (ESM), Figure ESM1. The mean scores were *M* = 21.61 (*SD* = 4.24) for perceived social support (ESSI), and *M* = 2.86 (*SD* = 1.47) for peer support. A total of *n* = 290 (79.45%) participants indicated having theoretical knowledge.

The average score on the LEC scale was *M* = 10.27 (*SD* = 8.44) for potentially traumatic events within the DLRG and *M* = 15.03 (*SD* = 14.20) for events outside. The two scores correlated at *r* =.36. A more detailed comparison revealed contextual differences in event frequency. Events such as sudden accidental death and the recovery of bodies were more frequently reported outside the DLRG, whereas physical or sexual assault was more commonly reported within the DLRG. A complete overview of potentially traumatic events by context is presented in the ESM (Table ESM1).

Screening of the LEC-5 open-ended question on particularly stressful events identified five common types: resuscitation attempts (54 mentions), incidents involving children (45), accidental deaths (37), recovery of bodies (31), and flood-related operations (27), including references to the recent “Ahrtal flood” in Germany. Some participants (19) described multiple events, despite being instructed to describe only one, and another 171 responses could not be classified into a larger category. Responses to other open-ended questions also indicated that many participants were involved in other aid organizations, such as the police, fire department, or military, through additional volunteer work or professional roles.

### Confirmatory analysis

The parameter estimates from all models described in the following sections are shown in Table 1. An investigation of Model 0, which included only a random intercept for predicting symptom severity and a constant zero-inflation component, revealed a non-negligible degree of clustering in PTSS scores across local groups, with ICCs of 0.64 for the full model and 0.22 for the count component. As described earlier, all subsequent models varied only in the count component.

**Table 1:** Regression coefficients with 95 % confidence intervals (*N* = 365)

Predictor	Model									
	0	1	2	3	4	5	6	7	8	9
Intercept: local group	.218 (.467)	.178 (.422)	.188 (.434)	.186 (.431)	.165 (.406)	.184 (.429)	.185 (.430)	.188 (.434)	.165 (.406)	.171 (.414)
Gender male		-.341*[-.652, -.030]	-.395* [-.702, -.089]	-.390* [-.697, -.084]	-.374* [-.679, -.070]	-.298* [-.567, -.028]	-.307* [-.577, -.038]	-.302* [-.572, -.032]	-.264 [-.530, .002]	-259 [-.524, .006]
Age		-.009 [-.021, .002]	-.014* [-.026, -.003]	-.014* [-.026, -.003]	-.018* [-.029, -.006]	-.019* [-.029, -.008]	-.018* [-.029, -.008]	-.018* [-.029, -.008]	-.012* [-.024, -.001]	-.013* [-.025, -.002]
LEC inside			.023* [.002, .044]	.022* [.001, .043]	.021 [-.017, .059]	.026* [.006, .045]	.027* [.007, .046]	.027* [.007, .046]	.031* [.012, .050]	.032* [.013, .051]
LEC outside			.009 [-.002, .021]	.011 [-.009, .031]	.009 [-.002, .021]	.007 [-.003, .017]	.007 [-.003, .017]	.008 [-.002, .018]	.009 [-.001, .019]	.010* [.000, .020]
Outside*Gender				-.003 [-.027, .021]						
Outside*Age				.000 [-.001, .001]						
Inside*Gender					-.004 [-.047, .039]					
Inside*Age					.002* [.000, .003]					
Social Support						-.079* [-.109, -.049]	-.079* [-.109, -.050]	-.079*[-.109, -.049]	-.078* [-.107, -.049]	-.076* [-.106, -.047]
Peer Support						-.109* [-.199, -.020]	-.107* [-.197, -.018]	-.112* [-.201, -.022]	-.100* [-.188, -.012]	-.091* [-.180, -.002]
Outside*Social Support							.001 [-.002, .003]			
Outside*Peer Support							-.002 [-.008, .004]			
Inside*Social Support								.002 [-.003, .006]		
Inside*Peer Support								.001 [-.010, .012]		
Rank-low									.477* [.123, .831]	.452* [.096, .807]
Rank-high									-.039 [-.587, .508]	-.081 [-.631, .469]
Prior knowledge yes										-.185 [-.487, .118]
conditional *R2*	.222	.225	.291	.291	.299	.426	.433	.424	.448	.458
marginal *R2*	.000	.049	.111	.111	.142	.250	.257	.242	.293	.299

*Note* Regression coefficients are reported with 95% confidence intervals in brackets with the exception of the variance of the random intercept, where standard deviation is reported instead. All reported estimates, including pseudo-*R²* values, refer to the count component

*LEC* Life Events Checklist, *LEC outside* Events outside the DLRG, *LEC inside* Events inside the DLRG. Rank was dummy coded with medium as the reference category

* *p* <.05

Adding the sociodemographic variables age and gender, assumed as influencing factors in H3a and H4a, as predictors significantly improved the model fit in Model 1 compared to the baseline (χ²(2) = 10.93, *p* =.004). Within this predictor set, gender was significantly associated with PTSS, whereas age did not reach statistical significance. The BF for the comparison between Model 1 and Model 0 was 0.65, indicating weak evidence in favor of Model 0 over Model 1. Nevertheless, as outlined earlier, model selection was based on significance testing, and Model 1 was retained for subsequent steps. In Model 2, predictors representing stressful events experienced inside and outside the DLRG context were added, corresponding to the influences hypothesized in H1 and H2. This addition further improved model fit (χ²(2) = 12.76, *p* =.002), with weak evidence (BF = 1.62) in favor of Model 2.

Model 3 tested interaction effects between stressful events outside the DLRG and sociodemographic predictors (corresponding to the effects hypothesized in H3b), but this did not significantly improve model fit (χ²(2) = 0.22, *p* =.898). Similarly, Model 4 included interactions with events inside the DLRG (corresponding to the effects hypothesized in H4b) and also showed no significant model improvement (χ²(2) = 5.16, *p* =.076) compared to Model 2. The corresponding Bayes factors were BF = 0.003 for Model 3 and BF = 0.04 for Model 4, providing strong evidence in favor of the more parsimonious Model 2, which included only main effects. Consequently, the interaction terms were not retained, and Model 2 remained the reference model.

Model 5 introduced perceived social support and peer support measures as additional predictors. Initially, these were included as fixed effects to compare against a model with random slopes for both predictors. The fixed-effects-only model significantly improved fit compared to Model 2 (χ²(2) = 38.02, *p* <.001, BF = 492,632.1). When random slopes were added to allow predictor effects to vary across clusters, the model exhibited convergence problems, likely reflecting small cluster sizes. In accordance with recommendations to simplify random-effects structures [69, 70], the random-effects covariance was fixed to zero. Even with this simplification, the estimated variances of the random slopes for both predictors were effectively zero. Both the likelihood-ratio test and Bayes factor favored the simpler version of Model 5, which included fixed effects for both predictors and only a random intercept as the random component (χ²(2) ≈ 0, *p* ≈ 1, BF = 0.003). This simpler model was used for all subsequent analyses.

In Model 6, interactions between perceived social support and stressful events outside the DLRG context were examined (corresponding to the effect hypothesized in H5 and H6), while Model 7 tested the equivalent interactions for events within the DLRG context. However, neither Model 6 (χ²(2) = 0.59, *p* =.745) nor Model 7 (χ²(2) = 0.73, *p* =.694) showed a significant improvement in model fit. In both comparisons, the Bayes factors strongly favored the simpler Model 5 (BF = 0.004 in each case). Consequently, the interaction terms were not retained, and Model 5 was identified as the best-fitting model prior to the exploratory analyses.

### Contextualization of hypotheses

To evaluate support for the hypotheses concerning variables and interactions, we considered model comparisons and the BF at the points when effects were introduced, along with the significance of each estimate conditional on all other included effects within the retained models. Regarding the influence of the frequency of potentially traumatic events inside (H1) and outside (H2) the DLRG, and thus the proposed building block effect, the comparison between Model 1 and Model 2 revealed a significant improvement in model fit, lending support to these hypotheses. However, the Bayes factor indicated only weak evidence in favor of the more complex model. Notably, the predictor representing stressful events inside the DLRG (LEC inside) consistently showed a significant positive association with PTSS in all models retained by the model comparison tests. In contrast, potentially traumatic events outside the DLRG (LEC outside) did not exhibit the expected associations within this predictor set. This finding lends further support to H1 while raising questions about the validity of H2.

The significant model comparison between Model 0 and Model 1 supports hypotheses H3a and H4a, although the Bayes factor did not corroborate this finding. Gender was significant across all confirmatory models. With male as the reference category, the negative association suggests that female emergency responders report higher PTSS, in line with the prediction of H3a. Age exhibited a significant negative association with PTSS in all models except the first, consistent with H4a, indicating that younger emergency responders tend to report higher PTSS levels. However, the inclusion of interaction effects between the frequency of potentially traumatic events and sociodemographic variables, as hypothesized in H3b and H4b and tested in Models 3 and 4, was not supported by model comparison tests. Bayes factors also provided strong evidence favoring models without these interactions. Therefore, hypotheses H3b and H4b were not supported by the present data.

The inclusion of additional influencing factors in Model 5, perceived social support and peer support, improved model fit, although random slopes were excluded due to small cluster sizes. This model received the strongest support across all comparisons according to the BF. Both support variables showed negative associations with PTSS severity, indicating that higher support corresponds to lower symptom severity. However, hypotheses H5 and H6, which concerned only interaction effects, were not supported. This is evidenced by non-significant model comparisons of Models 6 and 7 against Model 5, accompanied by very strong evidence from the BF against including the interactions.

### Exploratory analysis

Exploratory analyses revealed that adding the rank of the lifeguards in Model 8 significantly improved model fit compared to Model 5 (χ²(2) = 7.69, *p* =.02), with strong evidence in favor of the extended model (BF = 32.41). Within the predictor set, the contrast between low and middle ranks was significant, with participants holding a lower rank reporting significantly higher PTSS. Because of the reference category employed in the dummy coding, it can additionally be postulated that low rank is associated with greater PTSS compared to high rank. In contrast, the addition of theoretical knowledge in Model 9 did not lead to a further improvement in model fit, favoring the more parsimonious Model 8 (χ²(1) = 1.45, *p* =.229, BF = 0.11). Model 8 showed no significant association between gender and PTSS within the set of predictors, despite gender being consistently significant in all other models. To explore this discrepancy, we examined the relationship between gender and rank and found a correlation of γ = 0.65, indicating that these predictors likely explain some of the same variance.

In addition to the preregistered exploratory analysis, we investigated whether the type of event identified as the worst, as described in the descriptive statistics section, was associated with PTSS severity. From a descriptive standpoint, among the five identified non-mixed categories, witnessing death was associated with higher severity scores. However, a nonparametric comparison of the different types of worst events revealed no significant differences between event types (χ²(4) = 10.87, *p* =.198).

## Discussion

The present study aimed to investigate the effects of several influencing factors on PTSS among German volunteer lifeguards organized in the DLRG, using a joint model framework with a specific focus on the building block effect, meaning that cumulative exposure to potentially traumatic events influences posttraumatic symptomatology. Consistent with previous findings by Fien et al. [11] and Armstrong et al. [71], the results indicate an effect of the number of events on PTSS. Controlling for other predictors, stressful events outside the DLRG were not significantly associated with PTSS, whereas events within the DLRG were; including both event types in the model improved predictive accuracy. This contrasts with findings by Rooke and de Terte [12], who observed no effect of total trauma exposure, but only for experiences in the personal domain. According to Calhoun and Tedeschi [72], trauma experienced in one’s personal life can exert a stronger influence on core beliefs because of its greater emotional salience and personal significance, thereby intensifying cognitive disruption. In the present study, however, the distinction is less straightforward, as volunteer work could be considered part of both personal and occupational life. Moreover, traumatic events outside the DLRG likely comprise a mix of personal and professional experiences. Research from Germany indicates that more than half of volunteers are engaged in multiple service areas [73] p. 102 and LEC-5 responses in this study likewise show that many volunteer emergency responders also hold professional or additional voluntary roles in fields such as the military, fire departments, and ambulance services. This overlap may explain why events within the DLRG emerged as a clearer predictor in the present analysis and highlights the need for future research to distinguish more precisely between contexts in which traumatic events occur.

The influence of sociodemographic variables was observed, but they did not moderate the relationship between traumatic events and PTSS, suggesting that the impact of such events on PTSS did not depend on sociodemographic characteristics, in contrast to previous studies [12, 29, 30]. Regarding the direct effects, younger German lifeguards appeared particularly vulnerable, consistent with findings by Fien et al. [11] and Rooke and de Terte [12]. The mechanisms underlying this association remain unclear; one possibility is that older emergency responders benefit from greater life experience and greater awareness of available support networks [11]. Alternatively, a selective dropout effect may occur, with lifeguards experiencing PTSS symptoms leaving the organization and thus being underrepresented among older age groups in the sample. Similarly, although the association between female gender and higher PTSS has been replicated multiple times and PTSD prevalence rates are generally higher among women [74], the reasons remain poorly understood. Genetic predispositions and hormonal influences have been proposed as possible contributing factors [27].

A comparable pattern was observed for peer support measures. Although no moderation effects were identified, the negative association indicates that emergency responders who report higher levels of peer support and perceived social support tend to report lower PTSS, echoing robust evidence of their protective value. For instance, Wang et al. [75] demonstrated a reciprocal relationship between social support and PTSS in a meta-analysis of longitudinal studies, while Fjeldheim et al. [76] identified social support as a significant predictor of PTSD in paramedic trainees. Qualitative findings further suggest that peer support interventions can reduce stigma and promote treatment-seeking [77]. Moreover, the exploratory analysis considered additional potential influencing factors. In our sample, lower rank was positively associated with PTSS compared to higher ranks. One possible explanation is that emergency responders in lower-ranking positions are more frequently deployed directly to the scene due to operational structures, resulting in greater direct exposure to incidents than their superiors, who are often positioned further from the scene and have less direct interaction with individuals affected by the incident [78]. Thus, even when individuals of different ranks reported a similar number of potentially traumatic events, as controlled for in our model, the events for higher-ranking personnel may have occurred further in the past. This temporal distance could, in turn, help explain why higher-ranking personnel reported lower current symptom severity, as delayed onset of traumatic stress after exposure is a relatively rare phenomenon compared to immediate onset [79, 80].

In our sample, the presence of theoretical knowledge was not associated with PTSS, suggesting that this factor may not play a substantial role in influencing PTSS outcomes. This finding contrasts with prior evidence indicating that insufficient knowledge is linked to more negative mental health attitudes [81]. The lack of an association observed here may reflect the simplicity of the measure, which consisted of a single yes/no question, without information on the source of the knowledge, whether it was acquired before or after exposure to traumatic events, or how recently it was obtained. Prospective studies of educational measures would help clarify the temporal relationship between knowledge acquisition and mental health outcomes.

### Limitations and future directions

The present study relied on a convenience sample with self-selection to participate, resulting in a disproportionately high number of younger participants, which may have influenced the observed associations. A self-report bias may also have affected the results, particularly given the cultural context of first responder populations. Due to stigmatization and cultural norms, some participants may have been less forthcoming about the psychological impact of their work. Jones et al. [82] identified several barriers to help-seeking in emergency responders, including the belief that they are not allowed to show weakness, while Haugen et al. [83] highlighted the stigma surrounding mental health problems in this population. Moreover, as noted previously, lifeguards who develop more severe PTSD symptoms may be more likely to discontinue their volunteer work due to avoidance symptoms, introducing a selection bias and rendering them inaccessible to the present research. Ideally, future research should employ a longitudinal design, beginning with lifeguard training and following participants over time while also tracking quitting. A compelling example of this approach is provided by Gulliver et al. [84], who followed firefighters during their first years of service and assessed symptom development through regular interviews. Conducting the study on site within local groups could help to obtain a more representative sample, as self-selection bias may be reduced when the study is implemented in cooperation with the organization as a whole. A longitudinal design could also help clarify the temporal relationships between support measures, theoretical knowledge, and PTSS, thereby disentangling direct, indirect, and reciprocal effects. In particular, peer support and theoretical knowledge should be assessed using more than a single self-report item, as their measurement in the present study constitutes a limitation. Ongoing monitoring over time could facilitate more reliable assessment of these potential influencing factors. Additionally, diagnostic validity could be improved by using a clinical interview rather than relying solely on the PCL-5. The Clinician-Administered PTSD Scale for DSM-5 (CAPS-5) [85] is considered the gold standard for PTSD assessment. Furthermore, the adapted frequency-based administration of the LEC-5, including several additional items, has not been independently psychometrically validated, and comparability with studies using the standard LEC-5 format may be limited.

Statistical modeling, utilizing GLMMs, proved successful and allowed for a more differentiated investigation of correlates of PTSS in lifeguards than previous studies, thereby increasing confidence in the validity of the detected effects. However, the hypothesis testing focused only on the count component and did not examine the zero-inflation part of the model. This omission may help explain the absence of moderating effects, as earlier studies did not apply a zero-inflation approach, meaning their reported effects may represent a mixture of processes. Nevertheless, we were able to replicate several established correlations from the existing literature [5, 28, 31, 44]. A reanalysis of prior datasets, as well as new studies employing the GLMM approach, could help clarify the results and open new research avenues, particularly for identifying factors that predict a true non-zero value for PTSS (i.e., predictors in the zero-inflation part). Such analyses should also, as discussed earlier, incorporate a more detailed separation of when and where events occurred, acknowledging that many participants hold multiple roles as emergency workers — some in a volunteer capacity, others as part of their occupation — in addition to experiences from their private lives. To our knowledge, this is the first study in this context to apply mixed-effects modeling. Although our cluster sizes did not permit reliable estimation of random slopes, the high ICCs observed not only for the count component but also for the full model indicate substantial clustering by organizational units. Future studies could explore Level-2 predictors to explain random-intercept variance in both the zero-inflation and count components.

### Practical implications

The successful replication of the building block effect underscores the importance of the DSM-5’s recognition that multiple events can contribute to PTSD [49]. This insight suggests a need to revisit assessment tools, such as the PCL-5, which — despite being one of the few questionnaires revised for DSM-5 at the time — still focuses on a single “most stressful event” [49, 86]. While this approach may be appropriate in some clinical settings, it does not fully capture the complex and cumulative exposure experienced by lifeguards, who regularly face potentially traumatic events, or by similarly exposed groups such as other emergency workers or refugees. An alternative approach focusing on all events reported in an LEC-5, as used in the present study, or even on all events subjectively perceived as stressful by the individuals themselves, may be more valid.

The present study identified younger female lifeguards as a particularly vulnerable subgroup, suggesting that targeted training for this population may be warranted. Strengthening peer support measures remains important, as does providing educational courses that deliver structured theoretical knowledge going beyond symptom recognition to include preventive strategies, such as fostering strong social support networks outside the DLRG. Further awareness-building through focused seminars, especially for those in leadership roles, could benefit all emergency responders, helping to create an environment in which these topics can be addressed without fear or stigma.

## Conclusion

The present study contributes to understanding the factors influencing PTSS among lifeguards by examining a sample of volunteer lifeguards in Germany. By replicating the building block effect, it underscores the importance of addressing mental health challenges in volunteer settings, where cumulative exposure to traumatic events is common. The results highlight the protective roles of age, male gender, perceived social support, and peer support measures, suggesting practical directions for enhancing support systems. However, none of these factors moderated the relationship between traumatic events and PTSS. By examining stressful events occurring both within and outside the organization, the study provides insights into the specific challenges faced by volunteer lifeguards. The results should be interpreted with caution due to the cross-sectional design, and future research would benefit from longitudinal approaches to better understand symptom development and explore causal relationships.

## Supplementary Information

Supplementary Material 1.

## Data Availability

The present study was preregistered (10.17605/osf.io/dfe67). Participants consented to the sharing of study data only if no information enabling any form of identification was included. Consequently, certain demographic variables were excluded, which prevents replication of the analyses presented here. Nevertheless, all remaining study data and materials are openly available (https://osf.io/9wukf/files/osfstorage) and may be used for other analyses.

## Footnotes

1 For simplicity, the term “lifeguards” is used to refer to all DLRG emergency responders, although this designation does not necessarily reflect their level of training or certification.

2 Preregistration available at: 10.17605/OSF.IO/DFE67.

3 Documentation available at: https://osf.io/u3sm2.

## Abbreviations

BF: Bayes factor
BIC: Bayesian Information Criterion
CAPS-5: Clinician-Administered PTSD Scale for DSM-5
DLRG: German Life Saving Association (“Deutsche Lebens-Rettungs-Gesellschaft”)
DSM-5: Diagnostic and Statistical Manual of Mental Disorders-5
ESM: Electronic Supplementary Material
ESSI: ENRICHD Social Support Inventory
GLMM: Generalized linear mixed model
ICC: Intraclass correlation coefficient
LEC inside: Stressful events inside the DLRG
LEC outside: Stressful events outside the DLRG
LEC-5: Life Events Checklist for DSM-5
OSF: Open Science Framework
PCL-5: PTSD Checklist for DSM-5
PTSD: Posttraumatic stress disorder
PTSS: Posttraumatic stress symptoms
