# Supplementary material for: Posttraumatic stress in German volunteer lifeguards: evidence for the building block effect
Source: BMC Public Health. 2026 Jul 2;26:2023. doi: 10.1186/s12889-026-28126-1 (PMC13330046; doi:10.1186/s12889-026-28126-1)
Supplement: Supplementary file 1 — Supplementary Material 1. [file 12889_2026_28126_MOESM1_ESM.docx]

**Electronic supplementary material for
*Posttraumatic stress in German volunteer lifeguards: evidence for the building block effect***

**Figure ESM1***Full distribution of scale scores of the PCL-5*


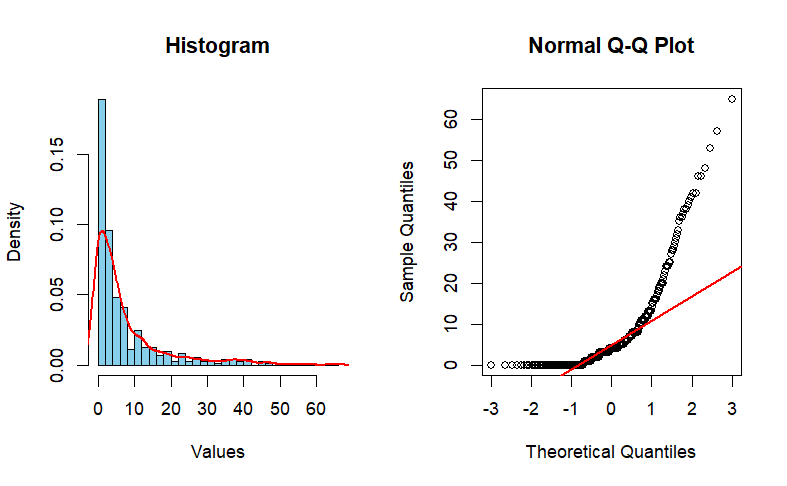


**Table ESM1***Frequencies and Means for each LEC Event Outside and Inside DLRG (N = 365)*

| **Event** | | **Frequencies** | | | | | | ***M*** |
| --- | --- | --- | --- | --- | --- | --- | --- | --- |
|  | | **0** | **1** | **2** | **3** | **4** |  |  |
| 01 | natural disaster |  |  |  |  |  |  |  |
|  | outside the DLRG | 152 | 124 | 54 | 19 | 16 |  | 0.97 |
|  | inside the DLRG | 121 | 135 | 80 | 20 | 9 |  | 1.07 |
| 02 | fire or explosion |  |  |  |  |  |  |  |
|  | outside the DLRG | 155 | 96 | 41 | 20 | 53 |  | 1.23 |
|  | inside the DLRG | 285 | 59 | 14 | 4 | 3 |  | 0.30 |
| 03 | transportation accident |  |  |  |  |  |  |  |
|  | outside the DLRG | 84 | 112 | 69 | 31 | 69 |  | 1.70 |
|  | inside the DLRG | 180 | 108 | 42 | 23 | 12 |  | 0.85 |
| 04 | serious accident |  |  |  |  |  |  |  |
|  | outside the DLRG | 160 | 96 | 40 | 19 | 50 |  | 1.19 |
|  | inside the DLRG | 214 | 95 | 37 | 12 | 7 |  | 0.64 |
| 05 | exposure to toxic substance |  |  |  |  |  |  |  |
|  | outside the DLRG | 247 | 66 | 24 | 11 | 17 |  | 0.59 |
|  | inside the DLRG | 316 | 41 | 7 | 1 | 0 |  | 0.16 |
| 06 | physical assault |  |  |  |  |  |  |  |
|  | outside the DLRG | 223 | 78 | 37 | 12 | 15 |  | 0.68 |
|  | inside the DLRG | 302 | 48 | 10 | 4 | 1 |  | 0.23 |
| 07 | assault with a weapon |  |  |  |  |  |  |  |
|  | outside the DLRG | 276 | 64 | 14 | 5 | 6 |  | 0.36 |
|  | inside the DLRG | 343 | 19 | 0 | 3 | 0 |  | 0.08 |
| 08 | sexual assault |  |  |  |  |  |  |  |
|  | outside the DLRG | 279 | 54 | 19 | 7 | 6 |  | 0.38 |
|  | inside the DLRG | 325 | 34 | 3 | 3 | 0 |  | 0.13 |
| 09 | other unwanted sexual experience |  |  |  |  |  |  |  |
|  | outside the DLRG | 239 | 77 | 22 | 14 | 13 |  | 0.59 |
|  | inside the DLRG | 280 | 62 | 17 | 4 | 2 |  | 0.32 |
| 10 | combat or exposure to a war zone |  |  |  |  |  |  |  |
|  | outside the DLRG | 314 | 32 | 10 | 1 | 8 |  | 0.24 |
|  | inside the DLRG | 358 | 4 | 1 | 1 | 1 |  | 0.04 |
| 11 | captivity |  |  |  |  |  |  |  |
|  | outside the DLRG | 359 | 5 | 1 | 0 | 0 |  | 0.02 |
|  | inside the DLRG | 364 | 0 | 0 | 1 | 0 |  | 0.01 |
| 12 | life-threatening illness or injury |  |  |  |  |  |  |  |
|  | outside the DLRG | 156 | 88 | 49 | 14 | 58 |  | 1.26 |
|  | inside the DLRG | 184 | 90 | 60 | 16 | 15 |  | 0.87 |
| 13 | rescue attempt of people in need of help |  |  |  |  |  |  |  |
|  | outside the DLRG | 148 | 92 | 45 | 18 | 62 |  | 1.33 |
|  | inside the DLRG | 76 | 118 | 87 | 46 | 38 |  | 1.59 |
| 14 | severe human suffering |  |  |  |  |  |  |  |
|  | outside the DLRG | 141 | 94 | 42 | 20 | 68 |  | 1.40 |
|  | inside the DLRG | 162 | 116 | 52 | 18 | 17 |  | 0.94 |
| 15 | sudden violent death |  |  |  |  |  |  |  |
|  | outside the DLRG | 204 | 88 | 25 | 10 | 38 |  | 0.88 |
|  | inside the DLRG | 247 | 70 | 32 | 9 | 7 |  | 0.52 |
| 16 | sudden accidental death |  |  |  |  |  |  |  |
|  | outside the DLRG | 224 | 71 | 30 | 11 | 29 |  | 0.77 |
|  | inside the DLRG | 151 | 112 | 63 | 21 | 18 |  | 1.02 |
| 17 | serious injury, harm or death you caused someone else |  |  |  |  |  |  |  |
|  | outside the DLRG | 342 | 16 | 5 | 0 | 2 |  | 0.09 |
|  | inside the DLRG | 355 | 5 | 2 | 1 | 2 |  | 0.05 |
| 18 | recovery of bodies |  |  |  |  |  |  |  |
|  | outside the DLRG | 265 | 46 | 22 | 12 | 20 |  | 0.56 |
|  | inside the DLRG | 165 | 113 | 57 | 15 | 15 |  | 0.91 |
| 19 | any other stressful event or experience |  |  |  |  |  |  |  |
|  | outside the DLRG | 193 | 103 | 34 | 13 | 22 |  | 0.82 |
|  | inside the DLRG | 223 | 103 | 29 | 5 | 5 |  | 0.54 |
